# Effect of New Water-Soluble Organosilicon Derivatives of Cartolin-2 on the Germination of Spring Common Wheat Seeds (*Triticum aestivum* L.)

**DOI:** 10.3390/ijms27010469

**Published:** 2026-01-01

**Authors:** Konstantin A. Kochetkov, Olga N. Gorunova, Nataliya A. Bystrova, Maxim S. Oshchepkov

**Affiliations:** 1 A.N. Nesmeyanov Institute of Organoelement Compounds of the Russian Academy of Sciences, Vavilov Str., 28, 119991 Moscow, Russia; 2 Faculty of Chemical and Pharmaceutical Technologies and Biomedical Drugs, D.I. Mendeleyev University of Chemical Technology of Russia, Miusskaya Sq., 9, 125047 Moscow, Russia

**Keywords:** plant growth regulators, water-soluble organosilicon compounds, cartolin-2, mesobiotic seeds spring wheat, germination, field tests

## Abstract

The development of innovative technologies aimed at increasing agricultural crop yields through the use of growth regulators that incorporate various biologically active chemical moieties is a key focus in modern global agroscience. In this context, novel water-soluble silicon-organic compounds derived from carbamate (**I**) and oxamate (**II**) have been synthesised by the introduction of an organo-silicone fragment into these biologically active molecules. The compounds are O-isopropyl-N-(2-trimethylsilyloxyethyl)carbamate (**III**) and O-isopropyl-N-(2-trimethylsilyloxyethyl)oxamate (**IV**). It has been found that the 1 × 10^−5^ M water solutions of the compounds **I**–**IV** exhibited growth-regulating activity on the seeds of spring common wheat (*Triticum aestivum* L.). Laboratory studies demonstrated that the new silicon-containing compounds **III** and **IV** had a positive influence on the following: the germination potential, the seed germination, the length of roots, and the growth and development of shoots. Field tests revealed that spring wheat treatment with the compounds **III** and **IV** yielded an augmentation in spike length, an elevated quantity of grains per spike, and a grain mass per spike in comparison to the control. The application of compounds **I**–**IV** resulted in a significant enhancement in spring wheat yield.

## 1. Introduction

The development of new technologies for increasing the yields of agricultural crops is a priority direction in modern agrochemical research. Modifications in fertilizers’ composition or seed pre-sowing treatment agents can significantly enhance crop yields.

Currently, the use of plant growth regulators (PGRs), primarily auxins [1,2,3] and cytokinins [4,5,6,7,8,9], has become increasingly prevalent to enhance seed germination and im-prove crop yields. Foliar application of auxin to spring wheat under field conditions im-proves its morphological characteristics: plant height, flag leaf area, spike length, quantity of spikelets, and grains per spike [1]. Cytokinins regulate cell division, meristem formation, photosynthesis, aging, uptake of macro- and microelements, and the response to biotic and abiotic stressors [10,11].

**Figure 1:**
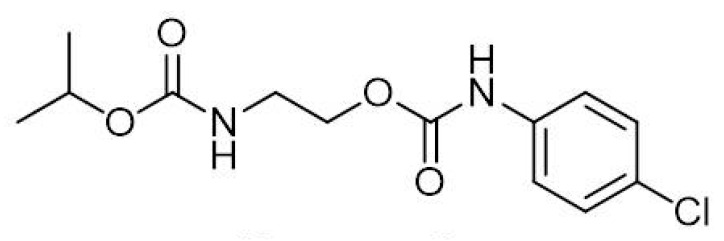
Structure of cartolin-2.

‘Cartolin-2’, N-(isopropoxycarbonyl)-O-(4-chlorophenylcarbamoyl)-ethanolamine (Figure 1) [5,12,13] is a structural analog of synthetic cytokinines and was developed as a universal agent necessary for risky farming regions [12]. It has been demonstrated that low doses of Cartolin-2 influence the growth and metabolism of higher plants. In addition, evidence suggests that it can inhibit chloroplast degradation, increase RNA polymerase activity, and slightly affect transpiration. As demonstrated in the findings of numerous trials, ‘Cartolin-2’ has been observed to exert a favorable influence on the yield in conditions of water deficit, low and high temperatures, limited lighting, soil salination, flooding, herbicide overdose, and the presence of phytopathogens [13]. However, the synthesis of ‘Cartolin-2’ involves the use of phosgene [12], which imposes significant restrictions on the organization of its production. Therefore, the intensive research and testing of the biological activity of its new derivatives, synthesized using safer and more technologically straightforward methods, are currently underway [5]. In preliminary assessments conducted in a controlled environment, the effects of compounds **I** and **II** on metabolic processes were investigated using tobacco cell cultures. The results demonstrated a clear, albeit variable, impact of these compounds on the metabolic processes under investigation [14]. Furthermore, it has been demonstrated that derivatives **I** and **II** regulate growth activity and exert a positive effect on spring wheat seeds [15].

On the other hand, it has been established that silicon compounds, such as PGR, promote plant growth, contribute to enhanced yield, facilitate plant adaptation to adverse environmental conditions, and augment protection against pathogens [16,17,18,19]. For instance, the application of silicon fertilizer has been demonstrated to enhance the resilience of rice stems, thereby mitigating the risk of lodging [16,17]. In a similar manner, maize exhibits enhanced drought tolerance and reduced susceptibility to stress when subjected to silicon fertilizer [18,19].

Nowadays, positive results concerning the application of hybrid compounds integrating multiple beneficial functions in agriculture have been reported [1]. Building on this progress, the aim of this research is to synthesize novel hybrid silicon-organic compounds by incorporating silicon-based fragments into biologically active carbamate and oxamate molecules. The potential ability of these newly developed hybrids to act as plant growth regulators will be evaluated through comprehensive laboratory and field experiments using the mesobiotic seeds of common spring wheat (*Triticum aestivum* L.).

## 2. Results and Discussion

**Scheme 1:**
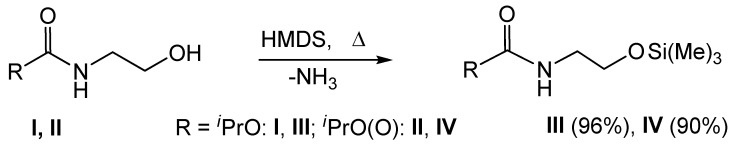
Preparation of organosilicone compounds **III** and **IV**.

Since the starting materials, i.e., O-i-Propyl-N-(2-hydroxyethylamino)carbamate (**I**) and O-i-Propyl-N-(2-hydroxyethyl)oxamate (**II**) are quite affordable, the synthesis of compounds (**III**) and (**IV**) in multi-gram quantities was not difficult. The compounds **III** and **IV** were obtained in a single stage with high yields according to the standard method for the preparation of trimethylsilyl derivatives of hydroxyl compounds by the action of hexamethyldisilazane (Scheme 1). New silicon-containing compounds **III** and **IV** have been found to demonstrate good solubility in water.

### 2.1. Laboratory Experiments

The development of growth regulators that combine various biologically active chemical fragments is one of the priority areas in the contemporary field of agricultural science. In a previous study [19], the growth-regulating activities of compounds **I** and **II** were investigated using the seeds of wheat that had been selected for germination in the 2019 harvesting season. It was shown that the compounds **I** and **II** softened the wheat seed coat, penetrated the grain, and activated physiological processes, which subsequently led to an increase in germination of 3–11% in comparison with the control samples. Furthermore, it was demonstrated that they had a stimulatory effect on the root length and shoot height of plants.

At the first stage, the germination of spring wheat seeds “Darya-2020“, treated by tested compound solutions of various concentrations was evaluated. According to the well-known technique [20], the tested compounds **I**–**IV** solutions were prepared in five concentrations from 1 × 10^−3^ to 1 × 10^−7^ M. Seeds treated with distilled water were used as a control. Germination potential and germination were calculated by formulas (Figures S1 and S2).

**Figure 2:**
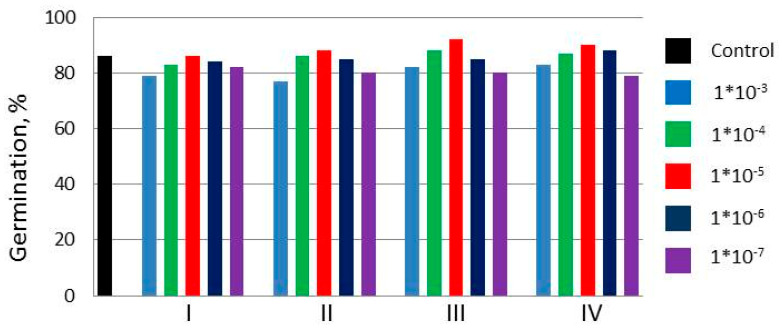
Germination of wheat seeds treated by the compounds **I**–**IV** solutions at a concentration of 1 × 10^−3^–1 × 10^−7^ M. The control was treated only by distilled water. Online calculator was used to analyze the data, which are presented as mean ± standard deviation. Statistical significance was determined by Student’s *t*-test based on *p* < 0.01 (**).

It was found that the compounds **I**–**IV** exhibited growth-regulating properties in comparison with the control, with the concentration of 1 × 10^−5^ M proving to be optimal (Figure 2).

Table 1 shows the results of the second stage of the studies of the tested compounds **III** and **IV**, in comparison with the compounds **I** and **II**, as well as with the known plant growth regulators: indoleacetic acid (IAA) and kinetin (Kin).

It is acknowledged that seeds stored over an extended period are susceptible to aging and a decline in germination potential (Gp). Gp is not a part of the germination process per se, but is an important component of pre-emergence growth of seedlings [21]. The root elongation rate (1–3 mm) is the principal feature of Gp [22]. Root elongation rate (1–3 mm) is the main feature of Gp [22]. Gp was determined 24 h after the start of the experiment in all seeds treated with compounds **I**–**IV**. Gp was greater than 50%, which was 10% higher than in the control.

The calculation seeds germination (G) was performed on the seventh day using the formula (Figure S2), as recommended by the International Seed Testing Association [23]. The investigation revealed that all compounds **I**–**IV** possess the capacity to regulate growth. Treatment of seeds with compound **III** resulted in the highest Gp of 59% and G of 95%, in comparison with the control sample (see Table 1).

Furthermore, the values for Gp and G, in the case of seeds treated with compounds **III** and **IV**, were higher than those for seeds treated with compounds **I** and **II**. The increase was 3% for Gp and 1–3% for G. A statistically significant relationship was identified between germination potential and germination (see Table S2), with a high positive correlation. Correlation between two or more variables is one of the most commonly used simple and straightforward statistical analytical methods in agriculture [24,25,26].

The process of seed germination is influenced by a combination of internal and external factors. It is evident that a multitude of extrinsic factors, including water, temperature, light, oxygen, and other environmental elements, exert a significant influence on the intricate process of seed germination. This stage is of pivotal significance in the plant cycle of development and reproduction.

It is acknowledged that silicon is classified as a quasi-essential element for plant growth and development. Silicon absorbed from solution by plant roots is transported to plant shoots, a process mediated by Lsi 1 (aquaporin-like transmembrane protein) [27]. In our work, we observed the rapid development of the root system. We hypothesize that Lsi 1 is expressed in the main and lateral roots. In addition, silicon can enhance the biosynthesis of endogenous cytokinins. However, the joint mechanism of interaction between silicon and phytohormones is not fully understood [28].

By the conclusion of the experiments (on the seventh day), active development of the root system was observed, which is required not only for the absorption of water and nutrients, but also to provide the plant with structural support.

The analysis revealed that plants treated with compounds **I** and **II** exhibited a total of five roots, each possessing a primary central root. This finding is consistent with the observed architecture of the root system of shoots whose seeds were treated with Kin [29]. The plants in the control group exhibited four roots each. Treatment of plant seeds with solutions **III** and **IV** resulted in the development of a highly branched root system, comprising six roots, each of which exhibited multiple root hairs. This phenomenon was analogous to the effect of IAA [30].

As asserted by the authors [31], plants exhibiting a pronounced root system are capable of thriving in the cultivation of agricultural crops under challenging climatic conditions.

The findings revealed that following seed treatment with compounds **III** and **IV**, the average root length exhibited a 28–50% increase compared to the control sample and a 4–14% increase when contrasted with the root length of seeds treated with Kin. Nevertheless, it was only the seeds that had been treated with the compound **III** that exhibited an average root length that was 6% greater than the root length of the seeds that had been treated with IAA.

It was noticed that the leaves of shoots, the seeds of which were treated with silicon-containing compounds **III** and **IV**, have a rigid and rough structure, compared with seeds treated with compounds **I**, **II** and control. According to Orzoł [32], organic silicon solutions help to increase the rigidity of leaves, which leads to the maintenance of shoots in an upright position for a long time.

Furthermore, 30% of the shoots that were treated with solution **III** exhibited the formation of a second leaf, which may suggest a high level of development.

The findings of this study demonstrated that the average height of shoots **I**–**IV** exhibited a 30% increase in comparison with the control. However, the shoots whose seeds were treated with the compound **III** exhibited a 5% increase in shoot height compared to the shoots germinated from seeds treated with Kin. Conversely, the average shoot height of the samples originating from seeds treated with compounds **II**–**IV** was found to be 6–12% higher than the shoot height of those germinated from seeds treated with IAA.

The average increase in biomass of all samples originating from seeds treated with solutions **I**–**IV** was found to be 18–25% higher than that of the control.

### 2.2. Field Test

Field tests were conducted in the Lipetsk region during the summer of 2024. The mesobiotic common wheat seeds of the “Darya-2020” variety were used for sowing work as well as for laboratory tests. This contemporary wheat brand demonstrates a high level of adaptability, exhibiting robust performance under diverse soil types and climatic conditions.

The mean air temperature in May was +12.5°, which is within the normal range for the growth of wheat seeds. The mean temperature in June was 20 °C, which is slightly above the mean for this time of year. Similarly, the mean rainfall was 67 mm, which is slightly above the mean for this time of year. These conditions were conducive to optimal photosynthesis and crop development. The month of July was characterized by warm weather, with an average temperature of 22 °C, and minimal rainfall, amounting to 24 mm. As demonstrated in [33], the findings of extensive research demonstrate a positive correlation between July precipitation levels and wheat yield.

The factors that were found to be most significant in determining yield formation in the experiment are as follows: the number of plants that survive until harvest, the density of the productive stem stand, and the productive business. The range of plant height was from 72 to 78 cm. In experimental plots, 216–231 plants per m^2^ were formed, accounting for 72–74% of the total sown seeds. In the control test, the number of plants was 204 per m^2^, indicating that the application of the tested compounds led to an increase in this indicator by approximately 5–13%.

It is important to note that the table presents average data on the number of productive stems. The application of foliar treatments comprising the tested compounds during the tillering stage resulted in the formation of plants exhibiting five to six productive stems (PS). Following treatment with compound **I**, 36% of plants exhibited 6 PS; with compound **II**, 31% exhibited 6 PS and 52% exhibited 5 PS; with compound **III**, only 37% formed 6 PS; and with compound **IV**, 66% exhibited 5 PS. The length of the spikes in the control group exhibited a significant difference (by 17–31%) in comparison to those in groups of samples treated with the compounds **I**–**IV**.

During the “tillering” period, spraying promotes the development of the root system, which increases the survival rate of shoots during subsequent growth stages. Spraying during the “steam elongation” stage leads to a more developed roots system, and the in-creasing stem thickness enhances resistance to lodging [20].

The yield of spring wheat was 0.54 kg/m^2^ in the control test. After the treatment with the tested compounds **I**–**IV**, the yield of spring wheat averaged between 0.59 and 1.05 kg/m^2^. The increase ranged from 0.11 to 0.57 kg/m^2^. The mass of 1000 grains in the control was 28.45 g, and the use of the studied compounds exceeded this indicator by approximately 5–11%.

**Figure 3:**
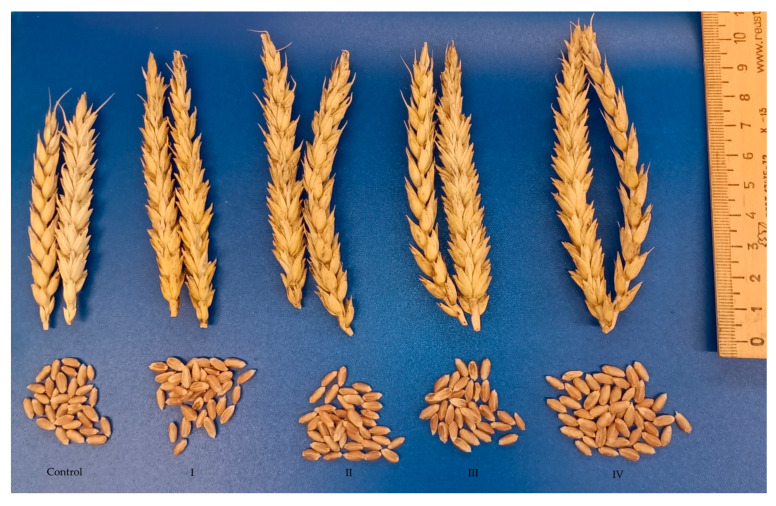
Type of spikes.

Figure 3 illustrated the part of the wheat crop 2024. It presents wheat ears, the shoots of which were treated with the tested compounds **I**–**IV** at the “tillering” and “stem elongation” stages, compared to the control. Control was not treated with tested compounds.

The tested compounds **III** and **IV** contributed to an increase in the spike length, the quantity of grains per spike, and the grain mass per spike in spring wheat compared to the control. The results of this study demonstrated that the application of tested compounds **I**–**IV** promoted an increase in wheat yield. The maximum yield increase was approximately twice as high in wheat treated with compound **II** compared to the control.

## 3. Materials and Methods

Since the starting compounds O-i-Propyl-N-(2-hydroxyethylamino)carbamate (**I**) and O-i-propyl-N-(2-hydroxyethyl)oxamate (**II**) were affordable [15] the syntheses of O-isopropyl-N-(2-trimethylsilyloxyethyl)carbamate (**III**) and O-isopropyl-N-(2-trimethylsilyloxyethyl)oxamate (**IV**) were not difficult and were carried out by us in multi-gram quantities. New compounds **III** and **IV** were obtained in one stage with high yields according to the standard technique [34].

New silicon-containing compounds **III** and **IV** have sufficient solubility in water to allow for testing of their biological activity. The structures of these compounds have been unambiguously established by a set of physico-chemical methods. The IR spectra contain bands characteristic of the trimethylsilyl group in the region of 839 and 1095 nm; in the ^1^H NMR spectra, in addition to signals characteristic of other fragments of compounds **I** and **II**, there is a singlet characteristic of this group at 0.1 ppm, and in the ^13^C NMR spectra there is a signal at −0.6 ppm.

### 3.1. Laboratory Test

Spring common wheat seeds (*Triticum aestivum* L.) “Darya-2020 [35], provided by LLC “Zhito,” were utilized in the laboratory experiments. At the commencement of the experiments in 2024, the seeds had been stored at +5 °C for a period of four years and were classified as mesobiotic in terms of lifespan [36].

Laboratory tests were performed according to the protocol described previously [1,20]. Four independent series of experiments using identical grow chambers with phyto-LED UFO lighting-79–01-00 with a wavelength of Red 615/Blu 457 nm with an intensity of at least 250 lux were carried out. The illumination of the samples was 12/12 h. The relative humidity of the air was 50%. The temperature was 20 °C. The duration of the experiment was 7 days.

### 3.2. Field Test

Field trials were conducted in the summer of 2024 in the settlement of Kuyman, Lebedyan District, Lipetsk Region, Russia (latitude 52°52′03″ N, longitude 39°16′58″ E). The Lipetsk region is part of the Central Chernozem region of agricultural crops in Russia. According to the Russian soil classification system, the term “black soil” primarily refers to the «Chernozems». These are humus-rich grassland soils, often found in the steppe and forest-steppe zones of Russia.

The total area of the site was 80 m^2^, and provided by LLC “Agro Expert Group.” The sowing scheme is presented in the Supplementary Material. The experimental plots measured 1 m^2^ each, with four replicates. Prior to sowing, no mineral fertilizers were applied. In the fall of 2023, mustard (*Sinapis alba*) was sown on the site as a cover crop. This plant does not fix nitrogen but is a fast-growing annual species that improves soil structure.

For each sown plot, 16.3 ± 0.02 mLm of 1 × 10^−5^ M solutions of the investigated compounds **I**–**IV** were prepared. The mesobiotic seeds of “Darya^®^”-2020 were manually sown at a rate of 300 seeds/m^2^ into the leached chernozem. The sowing depth was measured between 4 and 5 cm. The distance between rows was 12.5 cm. The field trials were conducted following the methodology described in [20]. Untreated seeds were utilized as the control group. Watering was conducted on a weekly basis using drip irrigation. During the growing season, weeds were manually removed. Foliar treatments were administered during the phases of tillering and stem elongation (S.V.). The experimental results are presented in Table 2.

### 3.3. Statistical Analysis

Statistical analysis of the results was performed using the “Statistica 10” software (StatSoft, Moscow, Russia). The data are presented as the mean ± standard deviation. Statistically significant differences from the control (water) at *p* ≤ 0.05 * and *p* ≤ 0.01 ** were identified according to Student’s *t*-test. The creation of graphs illustrating the results of the statistical analysis was achieved by utilizing the software programs Microsoft Excel 2010 and Corel Photo-Paint 12.

## 4. Conclusions

Water-soluble organosilicon compounds **III** and **IV,** being the derivatives of “Kartolin-2,” were obtained. These new compounds were synthesized according to standard methods. All tested compounds **I**–**IV** exhibited growth-regulating activity at the concentration of 1 × 10^−5^ M. Mesobiotic seeds treated with the tested compounds **I**–**IV** showed high germination potential and seed viability in laboratory experiments. It was noted that the tested compounds **I**–**IV** positively influenced root system development and shoot height, with an observed increase in green biomass. Our laboratory experiments did not identify a leading compound among the tested substances; therefore, field trials were conducted. The results demonstrated that the application of the tested preparations **I**–**IV** contributed to the increases in spike length, the number of grains per spike, grain mass per spike, 1000-grain weight, and wheat yield compared to the control. The compound **II** showed the best results with respect to the weight of grain per ear and yield kg/m^2^ and was identified as the most effective among the tested compounds.

## Figures and Tables

**Table 1:** Performance of wheat seeds treated with compounds **I**–**IV**, Kin and IAA.

	Gp %	G, %	Root Length, cm	Shoot Height, cm	Green Mass Gain in % Control
**Control**	40 ± 2.1	80 ±1.2	7.0 ± 0.8	11.5 ± 2.3	100
**I**	52 ± 2.2 *	90 ± 2.2 **	9.0 ± 0.8 **	15.8 ± 2.6 *	120 ± 2.8 **
**II**	56 ± 1.2 **	92 ± 2.8 *	9.7 ± 0.9 *	16.4 ± 3.2 **	123 ± 2.3 *
**III**	59 ± 1.2 **	95 ± 2.2 *	10.5 ± 1.1 *	17.2 ± 0.9 *	125 ± 2.9 *
**IV**	55 ± 2.2 **	93 ± 2.0 *	9.6 ± 1.5 *	17.4 ± 3.2 **	123 ± 3.6 **
**Kin**	58 ± 3.4 *	94 ± 2.5 **	9.2 ± 0.2 **	16.3 ± 0.2 *	124 ±1.6 *
**IAA**	51 ± 2.5 *	90 ± 3.8 **	9.9 ± 0.3 *	15.4 ± 0.4 **	118 ± 2.7 *

Germination potential (Gp, %), germination (G, %), root length (root, cm), shoot height (shoot, cm) and Green mass gain were determined. Seeds in a Petri dish treated only by distilled water were taken as control. Data are presented as mean ± standard deviation. The statistical significance was determined by Student’s *t*-test based on *p* < 0.05 (*) and *p* < 0.01 (**).

**Table 2:** The results of field tests conducted with the compounds **I**–**IV**.

	Gemination from the Site, %	Plant Height, cm	Quantity of Plant, pcs/m^2^	Quantity of Productive Stems, pcs/m^2^	Spike Length, cm	Quantity of Grains per Spike	Weight of Grain per Spike, g	Weight of 1000 Grains, g	Yield kg/m^2^	Increase g/m^2^
**Control**	68 ±1.73	72 ±1.15	204 ±3.46	816 ±3.64	8.0 ±2.02	31 ±1.81	0.77 ±0.015	28.45 ±0.41	0.54 ±0.07	-
**I**	72 ** ±1.32	72 ±0.57	216 ±5.19	864 * ±5.91	9.4 ** ±0.57	32 * ±1.54	0.74 ±0.026	30.13 ±1.10	0.85 * ±0.14	0.37 ±0.020
**II**	73 ** ±1.27	78 ±2.3	219 ±4.04	876 ** ±4.01	10.0 ** ±2.30	33 ±1.77	1.02 ** ±0.018	30.13 ±1.20	1.05 ±0.22	0.57 ±0.031
**III**	76 * ±1.72	73 ±1.73	228 ±2.30	912 * ±5.16	9.8 * ±0.77	32.5 ±1.34	0.78 * ±0.028	31.80 * ±1.16	0.59 ±0.15	0.11 ±0.021
**IV**	74 ±1.15	75 ±2.30	231 ±5.19	924 * ±4.05	10.5 ** ±1.73	35.5 * ±1.93	0.80 * ±0.031	30.96 ±1.34	0.73 ±0.12	0.25 ±0.07

Wheat seeds were treated with compounds **I**–**IV**. The control seeds were not subjected to any treatment. Data are presented as mean ± standard deviation. The statistical significance was determined by Student’s *t*-test based on *p* < 0.05 (*) and *p* < 0.01 (**).

## Data Availability

The original contributions presented in this study are included in the article. Further inquiries can be directed to the corresponding authors.

## Supplementary Materials

The following supporting information can be downloaded at: https://www.mdpi.com/article/10.3390/ijms27010469/s1.
