# Supplementary material for: Effect of New Water-Soluble Organosilicon Derivatives of Cartolin-2 on the Germination of Spring Common Wheat Seeds (Triticum aestivum L.)
Source: Int J Mol Sci. 2026 Jan 1;27(1):469. doi: 10.3390/ijms27010469 (PMC12786930; doi:10.3390/ijms27010469)
Supplement: Supplementary file 1 [file ijms-27-00469-s001.zip › ijms-3893369-supplementary.pdf]

Electronic Supplementary Information

S. I. Materials and Methods

S.I.1. Preparation of substances III - IV

O-i-propyl-N-(2-trimethylsilyloxyethyl)carbamate (**III**)

A 100 mL round bottom flask equipped with a reflux condenser was charged with 10 g of N-(2-hydroxyethyl)-O-isopropyloxamate (I) [34] (57 mmol) and 38.5 g (240 mmol) (50 mL) of HMDS. The reaction mixture was stirred on a magnetic stirrer at reflux until the evolution of ammonia ceased. At the end of the reaction (TLC control (IPA/EtOH/hexane 1:1:2, R_f ~0.87)), excess hexamethyldisilazane was distilled off in vacuum (65-70°C/3 mm). Purification was carried out on a silica gel column (ethyl acetate/petrol ether). 12.75 g of (**III**) were obtained, yield 90%.

^{1}H NMR (DMSO-d6, δ, ppm, J, Hz): 0.06 (s, 9H, $Si(CH_3)_3$); 1.26 (d, 6H, $CH(CH_3)_2$, J^3 = 6.24); 3.18-3.26 (q, 2H, NH-CH_2, J^3 = 6.11); 3.59 (t, 2H, CH_2OSi, J^3 = 6.11); 4.95-5.05 (m, 1H, CH); 8.75 (s, 1H, NH). ^{13}C NMR ($CDCl_3$, δ, ppm): -0.64 ($Si(CH_3)_3$); 21.51 ($CH(CH_3)_2$); 41.85 (NH-CH_2); 60.63 (CH_2-OSi); 71.38 (CH); 156.82 (O-C(O)); 160.07 (C(O)-NH).

IR (ν, cm^{-1}): 839.50 (0.431) ($Si(CH_3)_3$ def.); 1095.05 (0.405) (-C- O-Si val.); 1249.37 (0.296) (-C-O val.ether); 1523.39 (0.150) (NH-def. amide); 1687.88 (0.305) (-C=O val. ether сл.); 3316.69 (0.0349) (NH val.).

O-i-propyl-N-(2-trimethylsilyloxyethyl)oxamate (**IV**) was prepared similarly from oxamate **II**. Yield 96%.

^{1}H NMR (DMSO-d6, δ, ppm, J, Hz): 0.06 (s, 9H, $Si(CH_3)_3$); 1.14 (d, 6H, $CH(CH_3)_2$, J^3 = 6.24); 2.98-3.06 (q, 2H, NH-CH_2, J^3 = 6.11); 3.50 (t, 2H, CH_2OSi, J^3 = 6.11); 4.68-4.79 (m, 1H, CH); 6.96 (s, 1H, NH). ^{13}C NMR ($CDCl_3$, δ, ppm): -0.64 ($Si(CH_3)_3$); 22.14 ($CH(CH_3)_2$); 42.95 (NH-CH_2); 61.47 (CH_2-OSi); 67.89 (CH); 156.31 (O-C(O)). IR (ν, cm^{-1}): 838.86 (0.490) ($Si(CH_3)_3$ def.); 1092.10 (0.410) (-C-O-Si val.); 1248.94 (0.405) (-C-O val. ether); 1510.05 (0.173) (NH-def. amide); 1696.81 (0.264) (-C=O val.ether); 3343.63 (0.0422) (NH val.).

S.I.2. Instruments

^{1}H and ^{13}C NMR spectra were obtained using a Bruker DRX-400 NMR spectrometer at 400.13MHz (Germany). DMSO-d_6 (Aldrich) was used as a solvent and TMS as an internal standard. Chemical shift values were measured with an accuracy of 0.01 ppm; coupling constants are given in Hertz. IR spectra were recorded on an IR 200 Fourier-transform IR spectrometer (TermoNicolet, USA) with a resolution of 4 cm^{-1} (KBr). Reaction mixture composition analysis was performed by thin layer chromatography on silica gel (0.015–0.040 mm) aluminum backed TLC plates with an F254

fluorescent indicator (20×20 cm) (Merck Millipore, Darmstadt, Germany). “Kieselgel 60” (0.015–0.040 mm) silica gel (Fluka) was used for chromatographic separation on preparative scale.

S.II. Spectral data of compounds III and IV (Figures S1-S4)

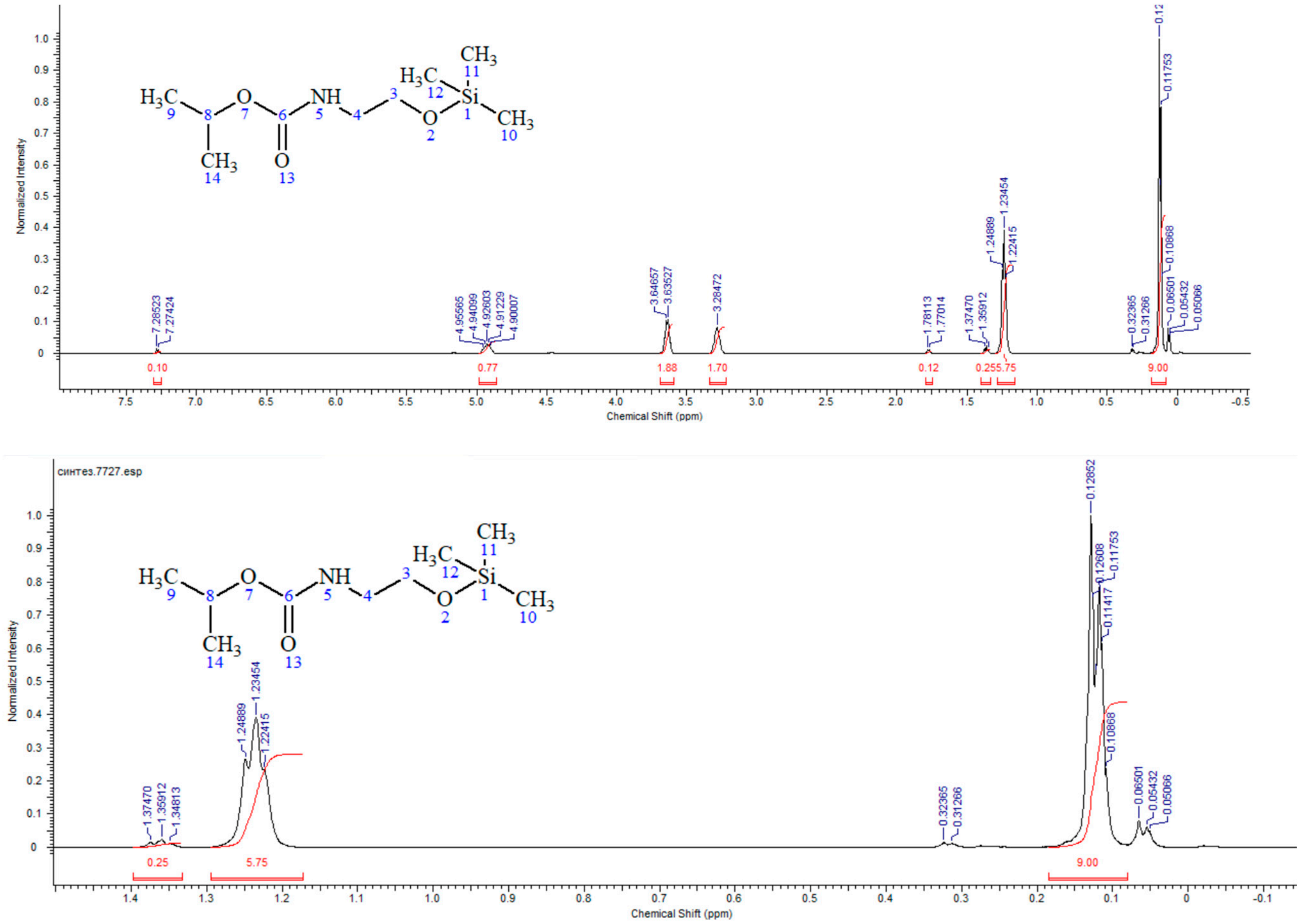

Figure S1. ^{1}H NMR spectrum of O-i-propyl-N-(2-trimethylsilyloxyethyl)carbamate **III**

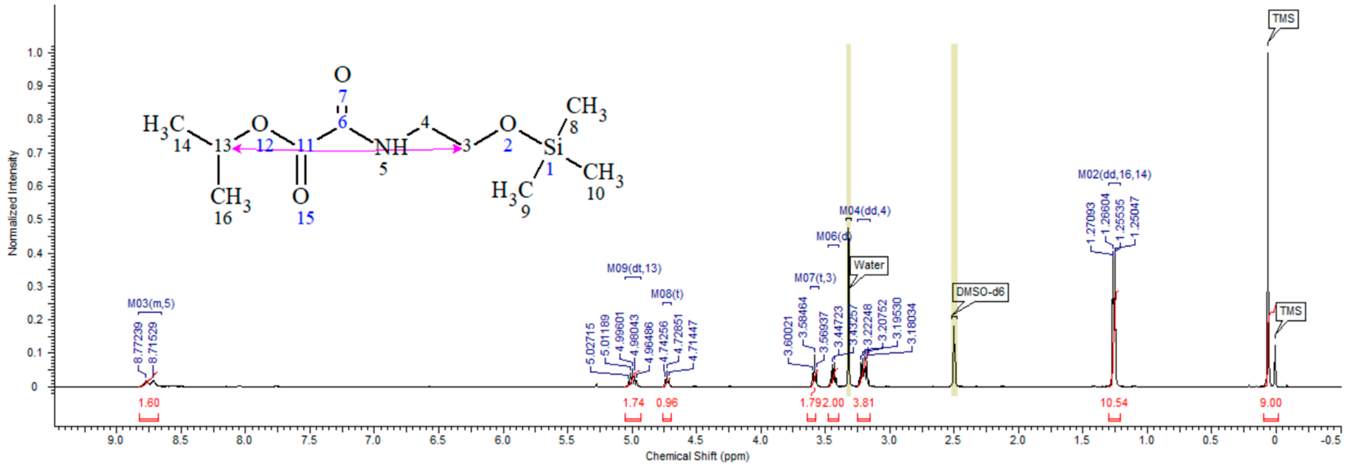

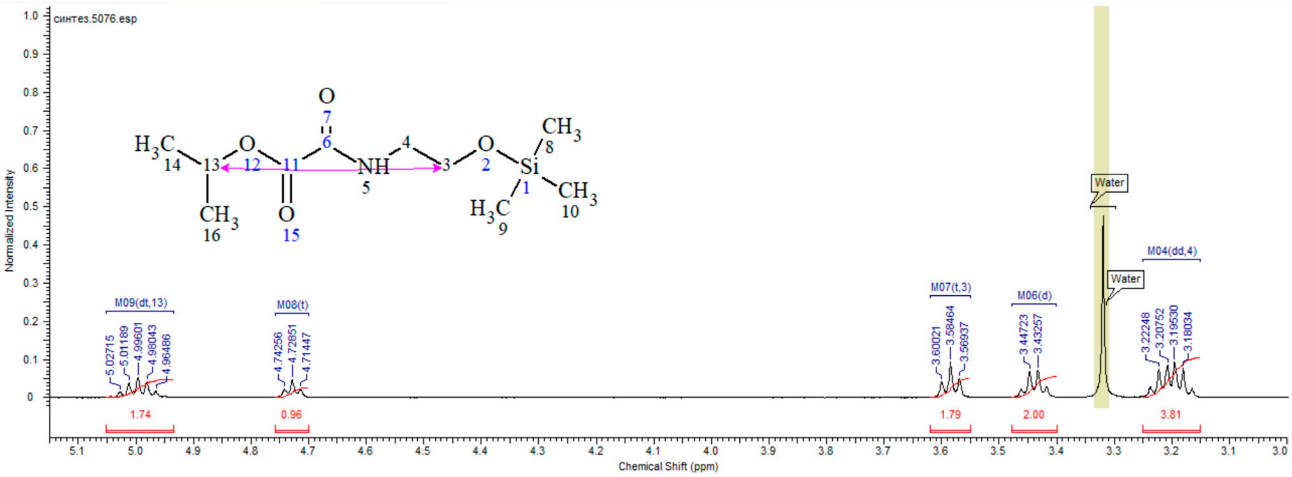

Figure S2. ^{1}H NMR spectrum of O-i-propyl-N-(2-trimethylsilyloxyethyl)oxamate **IV**

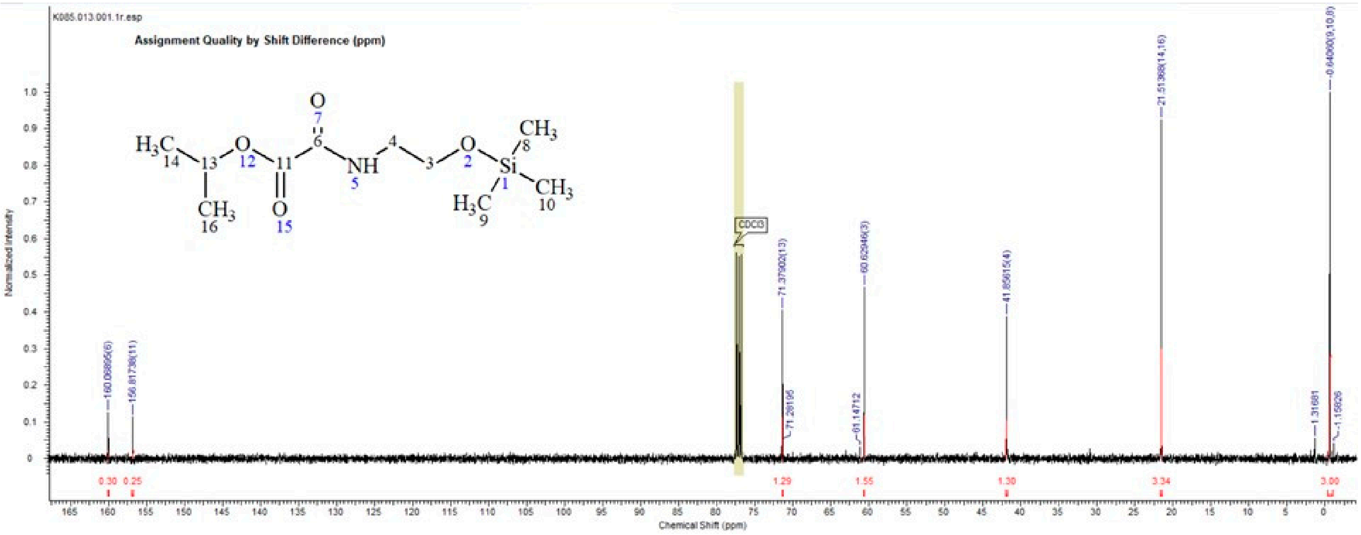

Figure S3. ^{13}C NMR spectrum of O-i-propyl-N-(2-trimethylsilyloxyethyl)oxamate **IV**

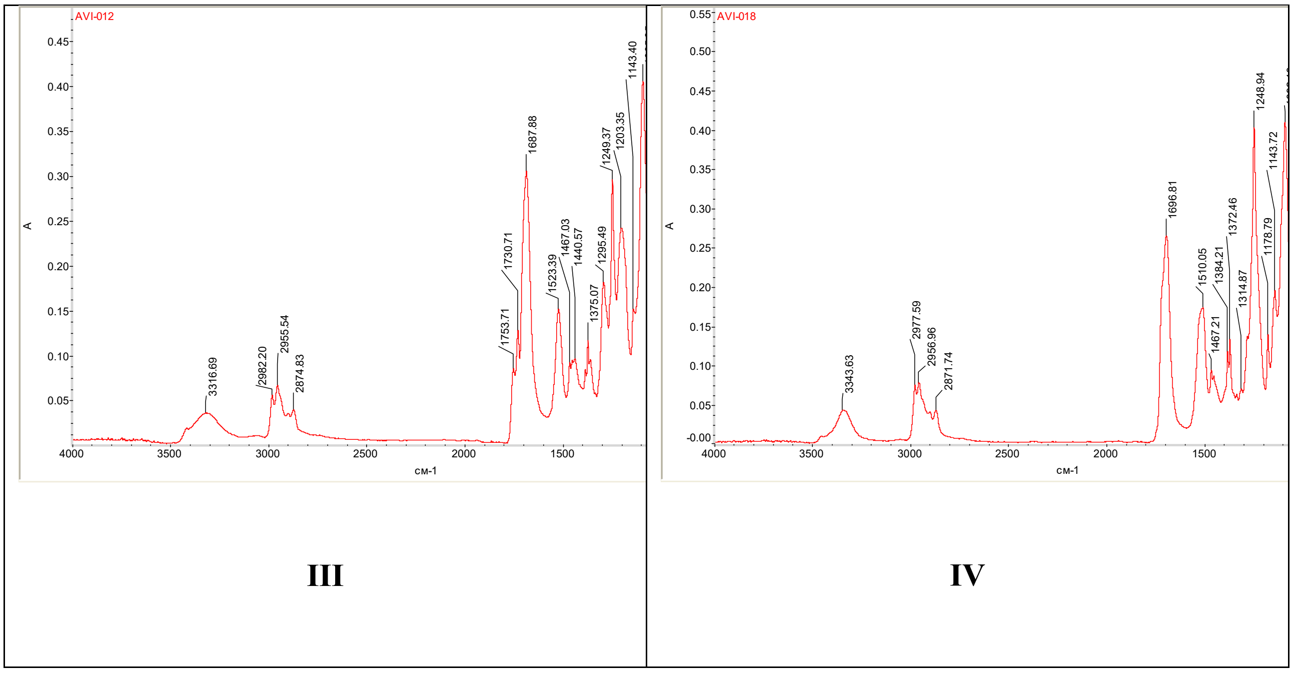

III **IV**

Figure S4 IR spectra O-i-propyl-N-(2-trimethylsilyloxyethyl)carbamate **III** and O-i-propyl-N-(2-trimethylsilyloxyethyl)oxamate **IV**

S. III. Laboratory experiments

Wheat seeds (*Triticum aestivum* L.) of the "Dariya®", crop 2020 was used. These seeds are registered in the State Register of Breeding Achievements of the Russian Federation No. 9705798 [28].

Before starting work, wheat seeds (*Triticum aestivum* L.) sterilized with 0.2% sodium hydrochloride solution for 10 minutes, washed three times with distilled water, dried at a temperature of 30 ° C for 48 hours. The dried seeds were stored at a temperature of 5 °C.

S. III.1. Methodology of the experiment

Processing of the main parameters of the laboratory test was carried out according to the formulas used in previous works [2, 29].

Four independent series of experiments in identical cameras with phyto-LED UFO lighting-79-01-00 with a wavelength of Red 615/ Blu 457 nm with an intensity of at least 250 lux were carried out. The illumination of the samples is 12/12 hours. The relative humidity of the air was 50 ± 2%. The temperature is 21 ± 1°C. The duration of the experiment is 7 days.

Fifty (50) pieces of dry sterilized seeds were placed on filter paper in rectangular Petri dishes 75 × 85 (mm) and treated by spraying with the studied compounds accordance with the test protocol. Wheat grains were treated with 0.3 ± 0.04 mL of compound solutions I-IV.

Vertical spraying was carried out in an isolated box with disposable screen. After spraying, the screen was removed. The surface of the box was disinfected and created with paper napkins. After spraying, the seeds were covered with filter paper and 10 ml of distilled water was poured. Then the Petri dishes with a lid were moved to the growth chamber. The first 24 hours of the experiment were conducted in the dark. The seeds were aired every day. Petri dishes were opened for 25 minutes, and 5-10 ml of distilled water was added so that the seeds did not dry out. On the third day of the experiment, the lids of the Petri dishes were removed so that the shoots grew.

The germination potential of wheat seeds was determined 24 hours after the start of the experiment according to formula *S1*:

Germination potential (%) = [Number of germination seeds 1d / Number of total seed] x 100 *(S1)*

Seed germination was calculated by formula *S2* [23] after the end of the experiment:

Germination (%) = [Number of germination seeds 7 d / Number of total seed] x 100 *(S2)*

Table S1. Results of preliminary work

		Gp<br>%	G,<br>%	Root Length, cm	Shoot height, cm	Green mass gain in % control
**Control**	0	52	86	6,5	14	100
**I**	$1x10^{-3}$	49	79	8,2	15,1	114
	$1x10^{-4}$	52	83	8,7	15,4	121
	$1x10^{-5}$	**54**	**86**	**8,8**	**16,6**	**122**
	$1x10^{-6}$	50	84	8,6	16	120
	$1x10^{-7}$	48	82	8	15,9	115
**II**	$1x10^{-3}$	48	77	8,1	16,1	115
	$1x10^{-4}$	52	86	8,2	16,4	123
	$1x10^{-5}$	**55**	**88**	**8,6**	**17**	**124**
	$1x10^{-6}$	51	85	8,3	16,8	120
	$1x10^{-7}$	45	80	7,9	15,6	113
**III**	$1x10^{-3}$	42	82	8,1	15,2	112
	$1x10^{-4}$	50	88	8,6	16,4	123
	$1x10^{-5}$	**55**	**92**	**9,1**	**17,3**	**125**
	$1x10^{-6}$	48	85	9	15,8	121
	$1x10^{-7}$	43	80	8,8	14,6	111
**IV**	$1x10^{-3}$	46	83	8,8	14,9	112
	$1x10^{-4}$	50	87	9,2	16,4	122
	$1x10^{-5}$	**52**	**90**	**9,6**	**17,2**	**123**
	$1x10^{-6}$	48	88	9,4	16,6	120
	$1x10^{-7}$	42	79	9	16,1	115

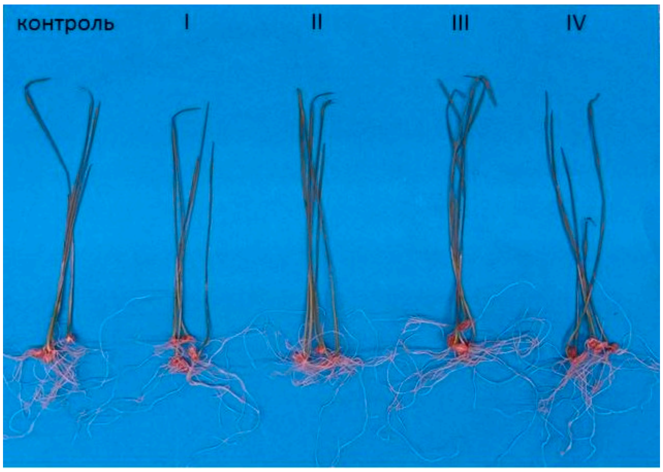

Figure S5. Results of laboratory research of compounds I – IV.

S.III.2. Laboratory Experiment

Aqueous solutions with concentrations of $1*10^{-3} – 1*10^{-7}$ M were prepared for tasted compounds **I – IV** according to a known technique [1, 20].

Wheat seeds (*Triticum aestivum* L.) of the "Darya® ", crop 2020, provided by LLC "Zhito", Oktyabrsky district, Ryazan, Russia (54.609836° S.w., 39.80188° V.D.) were used. These seeds are registered in the State Register of Breeding Achievements of the Russian Federation No. 9705798.

Before starting work, wheat seeds (*Triticum aestivum L.*) sterilized with 0.2% sodium hydrochloride solution for 10 minutes, washed three times with distilled water, dried at a temperature of 30 ° C for 48 hours. The dried seeds were stored at a temperature of 5 ºC.

S. IV. Pearson correlation analysis.

Correlation is a statistical procedure for quantifying the relationship or "consistency" between two variables. It is one of the most commonly used simple and straightforward statistical methods for analysis used in agriculture [24, 25]. Correlation coefficients for germination potential, germination and root length, stem height and green mass gain are shown in Table S2.

Table S2. Pearson correlation coefficient of agronomic traits (characteristics)

	**Germination potential**	**Germination**	**Root length**	**Shoot height**	**Green mass gain in % control**
**Germination potential**	**1**				
**Germination**	**0.962***	**1**			
**Root length**	**0.380**	**0.552**	**1**		
**Shoot height**	**0.702**	**0.795**	**0.420**	**1**	
**Green mass gain in % control**	**0.986***	**0.926***	**0.280**	**0.732**	**1**

* ($p \le 0.05$), ** ($p \le 0.01$).

Significant correlation between different agronomic characteristics has been found. Strong (r ≥ 0.7) and weak (r ≤ 0.3) correlations were denoted according to the correlation coefficient values. Correlation coefficient values between 0.3 and 0.7 (0.3≤ r≥ 0.7) were designated as moderate correlation [26].

The result of the analysis, a very high positive correlation between Gp and G (r=0.962), Gp and Gain (r=0.986), G and Gain (r=0.926) was found. Other traits such as Gp and S (r = 0.702), G and S (r=0.795), S and Gain (r=0.732) showed a high positive correlation. The most moderate positive correlation was between R and Gain (r = 0.280). Correlation analysis was performed using

the online calculator of correlation-regression analysis. The system is deterministic and is described by the linear equation y = c + ax.

V. S. Field Experiment

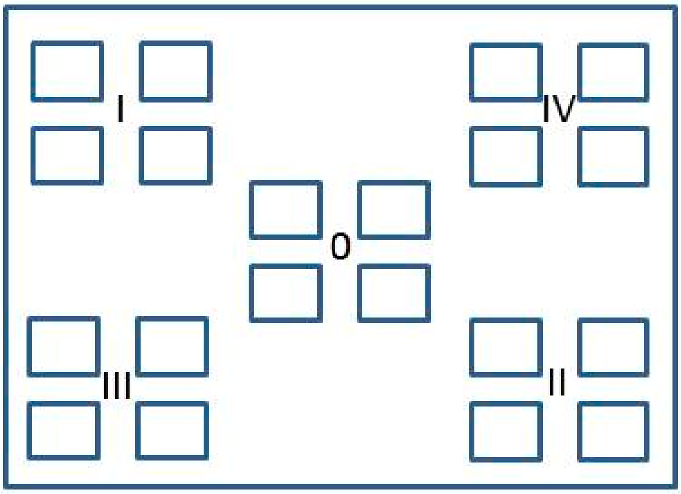

Figure S6. The scheme of the field diagram is a rectangle that was divided into squares. The width of the aisle and the width inside the four plots are 0.4 m. The total area of the plot is 8.8 x 8.8 = 77.44 m2.

Table S3. Meteorological data of the growing season 2024.

Months	Temperature			Precipitation		Hydrothermal coefficient
	Average daily ,°C	Average-Multi year ,°C	deviated from the norm, +, -, °C	Total, mm	Average annual, mm	
**May**	12.2	14.9	-2.2	16	47	1.31
**June**	20.4	18.4	+2,0	67	62	3.24
**July**	22.9	20.5	+2.4	24	68	1.05
**August**	20.5	19.0	+15	24	55	1.17

The hydrothermal coefficient (HTC), also known as Selyaninov's hydrothermal coefficient, is a ratio used to assess the moisture conditions of a region, particularly in agricultural and industrial forestry. HTC is calculated using the formula: $K = R*10/\Sigma t$; where R is the sum of precipitation in millimeters over a period with temperatures above +10°C, Σt is the sum of temperatures in degrees Celsius (°C) over the same time. This coefficient helps determine if a region is adequately humid, excessively humid, or dry.

On the day of sowing (May 5, 2024), the weather was sunny in the morning, with a temperature of +8°C and humidity at 40 %. The wind direction was from the west, shifting to north-west.

The meteorological conditions in the first decade of May were extreme: there were minor frosts on the proposed site, which did not affect the germination of spring wheat, as the soil temperature of +5°C was optimal for emergence. Precipitation amounted to 34% of the norm, and the average temperature in May was +8.8°C during the second decade, positively influencing the uniform emergence of the crop.

Wheat phenological stages refer to the observable stages of development in a wheat plant's life cycle. These stages are crucial for understanding growth, optimizing agricultural practices, and predicting yield. Common stages include germination, tillering, stem elongation, booting, heading, flowering, and maturity.

The foliar treatment of wheat tillers during the tillering and stem elongation phases occurred under relatively favorable conditions. The temperature regime in the first and second decades of June was characterized by moderately warm conditions with sufficient moisture, which contributed to the formation of additional stems in the plants. The average monthly temperature in June was 20.4°C, and 67 mm of precipitation fell (108% of the norm).

June 4 was sunny day of foliar processing during the tillering phase. The air-temperature was 20 °C. June 19 was sunny day of foliar processing during the stem elongation stage. The air temperature was 22 ºC.

During the "tillering " period, spraying promotes the development of the root system, which increases the survival rate of shoots during subsequent growth stages. Spraying during the "stem elongation" stage leads to a more developed roots system, enhances resistance to lodging by increasing stem thickness.

July was characterized by moderately warm temperatures with minimal precipitation (a total of 23 mm, 35% of the norm). IT is consistent with long-term average values. Full grain ripeness was reached in the third decade of July. During this period, the weather was warm with moderate humidity; the air temperature was 22°C, and 0.7 mm of precipitation fell.

The crop took place on August 10, 2025. By 9 a.m., the sun had risen, the temperature was +22°C, and the wind direction was from the west.
